# The treatment dilemma in adult patients with peripheral pulmonary artery stenosis of diverse etiologies

**DOI:** 10.1186/s43044-021-00190-5

**Published:** 2021-07-15

**Authors:** Zahra Hosseini, Ata Firouzi, Bahram Mohebbi, Ehsan Khalilipur, Mohammadreza Baay, Kiara Rezaei Kalantari, Iman Harirforoosh, Zahra Khajali

**Affiliations:** 1 Cardiovascular Intervention Research Center, Rajaie Cardiovascular Medical and Research Center, Iran University of Medical Sciences, Tehran, Iran; 2 Rajaie Cardiovascular Medical and Research Center, Iran University of Medical Sciences, Vali-Asr Ave, Tehran, 1996911101 Iran

**Keywords:** Peripheral pulmonary artery stenosis, Diagnostic modalities, Treatment approach, Endovascular intervention, Balloon angioplasty, Stenting

## Abstract

**Background:**

Peripheral pulmonary artery stenosis (PPAS) is a rare and underdiagnosed phenomenon that is reported infrequently in adult patients. Most patients with PPAS have concomitant congenital heart diseases, a history of palliative surgical therapies during childhood, or syndromic characteristics. Acquired cases are rare, and they are underestimated in adulthood and managed inappropriately.

**Case presentation:**

This case series describes 3 adult patients with PPAS of diverse etiologies and discusses their underlying causes, diagnostic modalities, and treatment strategies.

**Conclusions:**

In patients with PPAS, sufficient heed should be paid to endovascular interventions such as balloon dilation and primary or bailout stenting, not least vis-à-vis the type and size of balloons or stents as well as complications and preventive strategies.

## Background

Peripheral pulmonary artery stenosis (PPAS) is a rare and underdiagnosed condition that is seldom reported in adulthood. The first case report was published in 1938, since which time the process of its diagnostic development and categorization has led to Gay’s classification based on coarctation types [1, 2]:

I. Coarctation of the main pulmonary artery (MPA) or the right (RPA) or left (LPA) pulmonary artery
II. Coarctation at the bifurcation of the MPA extending into the RPA and the LPA
III. Multiple peripheral coarctations
IV. A combination of main and peripheral coarctations

In the last classification of pulmonary hypertension (PH), PPAS is categorized as part of group IV. Misdiagnosis as idiopathic PH or chronic thromboembolic pulmonary hypertension (CTEPH) is not uncommon [2, 3]. PPAS can manifest itself as an isolated congenital or acquired disease or more frequently as part of complex congenital heart diseases or syndromic circumstances. Isolated congenital cases are reported sporadically, while acquired cases are reported in vasculitis diseases such as Takayasu’s arteritis or Behçet’s disease or after palliative surgeries such as the modified Blalock-Taussig shunt, the Waterston shunt, PA banding, and patch augmentation of the central PA or RV-PA conduits [2–4]. The most frequent congenital heart diseases with concomitant PPAS are the tetralogy of Fallot, the double-outlet right ventricle (RV), the patent ductus arteriosus, the atrial septal defect, and the pulmonary atresia–ventricular septal defect. Most often, they are diagnosed in syndromic cases such as Williams–Beuren syndrome, Alagille syndrome, Noonan syndrome, congenital rubella syndrome, Ehlers–Danlos syndrome, cutis laxa, and Russell–Silver syndrome [5].

The hemodynamic effects of PPAS are dependent on the number and location of the stenosis, the severity of the obstruction, and concomitant congenital anomalies. PPAS in adults are predominantly located in the bilateral main branches or the lobar pulmonary arteries; nonetheless, in some cases, serial segmental involvement has been reported [6].

In isolated types in adults, the range of symptoms is highly variable. Patients may be asymptomatic or symptomatic following long-term RV pressure overload, RV hypertrophy, or diastolic dysfunction. On physical examination, according to the location and severity of the stenosis, peripheral bruits or continuous murmurs over the back or on either lateral side of the chest and a loud P2 in the left second intercostal space might be audible. Additionally, RV heave and elevated jugular venous pressure are pronounced, with hepatomegaly, ascites, and peripheral edema manifesting themselves in advanced cases.

## Case presentation

### Case 1

A 37-year-old-man with a history of progressive exertional dyspnea during the preceding 6 years was referred to our clinic for further evaluation of PH. The patient’s history revealed surgery for keratoconus and inguinal hernia. Furthermore, he reported a family history of surgery for keratoconus and uterine prolapse in his sister. Physical examination revealed normal peripheral pulses and blood pressure in all extremities. He had soft and hyper-elastic skin as well as atypical facial features, consisting of down-slanting palpebral fissures, a beaked nose, and prominent upper lips. A third-degree systolic ejection murmur and a loud P2 in the left second intercostal space could be heard. Electrocardiography indicated an indeterminate axis and evidence of RV hypertrophy. In chest X-ray, reduced pulmonary vascularity was prominent.

Transthoracic echocardiography (TTE) and transesophageal echocardiography showed a normal-sized left ventricle (LV) with mild systolic dysfunction, moderate-to-severe RV enlargement with moderate dysfunction, and severe RV hypertrophy. No valvular, subvalvular, or supravalvular pulmonary stenosis was denoted. Moderate pulmonary insufficiency, moderate tricuspid regurgitation (gradient = 135 mmHg), severe narrowing in the distal part of the LPA (pressure gradient = 100 mmHg), dilation in the proximal part of the coronary arteries, and a large and stretched patent foramen ovale (6 mm) with bidirectional shunting were reported.

**Fig. 1:**
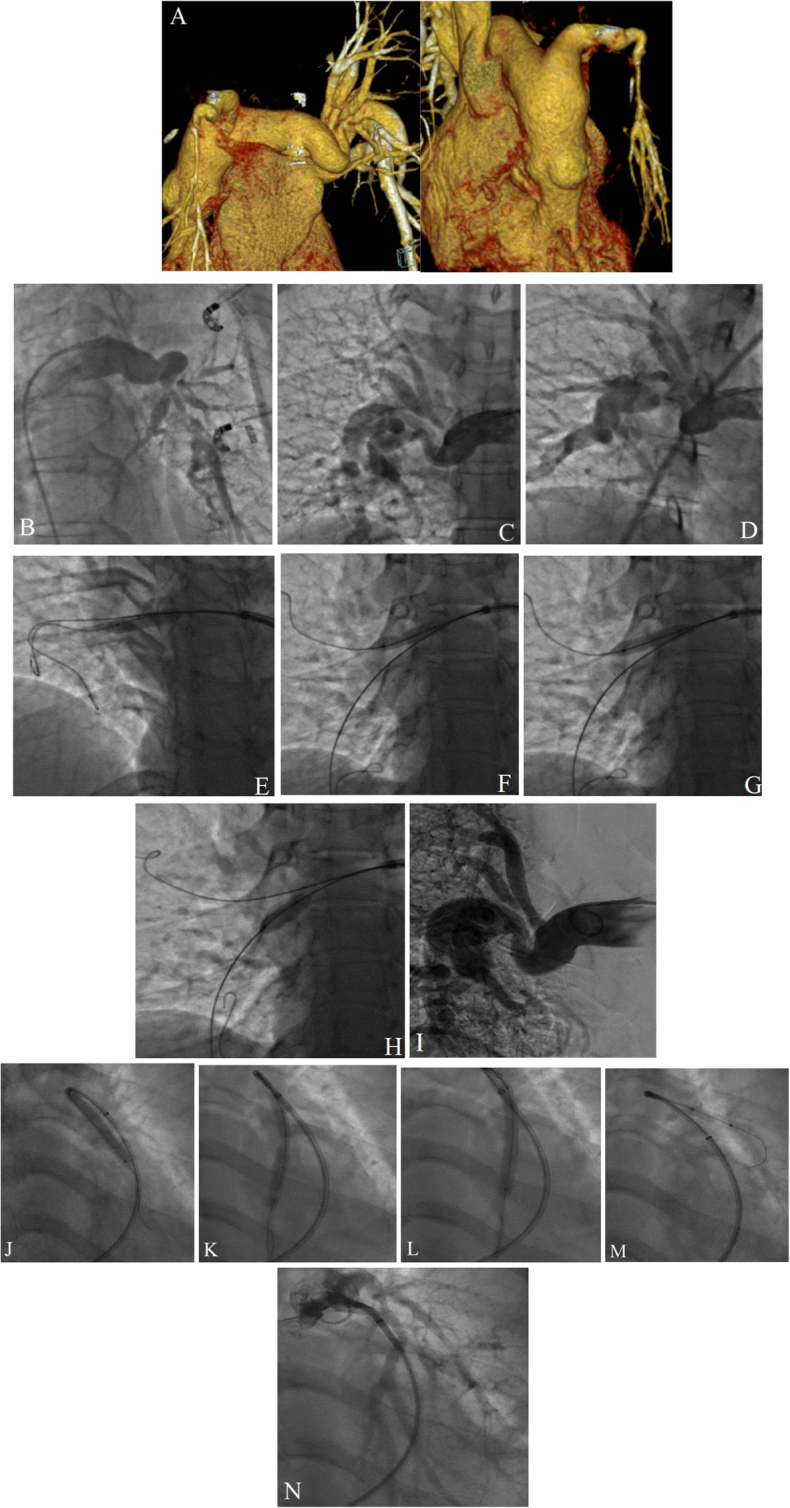
(**A**) The images illustrate a pulmonary computed tomography angiography, showing bilateral peripheral pulmonary artery stenosis. (**B**, **C**, and **D**) The images illustrate the left pulmonary artery injection, showing significant stenosis in the upper, middle, and lower branches (**B**). The right pulmonary artery injection reveals severe stenosis in the upper and lower lobar branches (**C** and **D**). (**E**, **F**, **G**, **H**, and **I**) In the first session, through a long delivery sheath and a NaviCross Support Catheter, the lower and middle branches of the right pulmonary artery are wired with 0.014-in coronary wires. They are then replaced with Amplatz Super-Stiff guidewires. Balloon percutaneous angioplasty is performed on the lower lobar branch of the right pulmonary artery (**E**). Afterward, sequentially, balloon percutaneous angioplasty is carried out on the middle segmental branch (**F** and **G**) and the lower segmental branch (**H**) successfully with a mild residual stenosis (**I**). (**J**, **K**, **L**, **M**, and **N**) In the left pulmonary artery, primary balloon percutaneous angioplasty is performed on the lower lobar artery (**J**). The other lower branch is wired before balloon percutaneous angioplasty is conducted. The central waist completely disappears (**K** and **L**). Finally, the procedure is completed with balloon percutaneous angioplasty on the upper branch (**M**) with perfect results (**N**)

A cardiac and pulmonary computed tomography angiography (CTA) was performed to evaluate the locations, numbers, and diameters of the distal branches precisely. It delineated multiple segments of sequential stenosis and dilation in the distal parts of both pulmonary arteries (Fig. 1A).

Given the patient’s symptoms and echocardiographic and CTA findings, he was scheduled for catheterization, right heart catheterization (RHC), and probably therapeutic interventions.

#### Right heart catheterization data (Table 1)

##### Procedural technique

Under local anesthesia, hemodynamic monitoring, and complete intravenous heparinization, vein sheaths (6 F) were inserted into the right femoral artery. A selective coronary angiography showed aneurysmal dilation in the left main artery and the proximal part of the left anterior descending artery. Selective LPA and RPA injections in the anteroposterior projection illustrated significant stenosis in the upper, middle, and lower branches of the LPA and significant stenosis in the middle and lower lobar branches of the RPA with segmental aneurysmal dilation in the distal parts (Fig. 1B, C, and D). Initially, through a NaviCross Support Catheter, the lower branch of the RPA was wired with a 0.014-in coronary workhorse wire. Next, the catheter was advanced over the wire. Then, the wire was replaced with an Amplatz Super-Stiff guidewire, and another Amplatz Super-Stiff guidewire was inserted in the middle branch. Thereafter, balloon percutaneous angioplasty (BPA) was performed on the lower lobar branch (at the site of the bifurcation of the lower and middle segmental arteries) with a 5.5 × 20 Aviator, a 6 × 30 Ever-Cross, and an 8 × 30 Ever-Cross, respectively (Fig. 1E). Subsequently, BPA was performed on the middle (Fig. 1F and G) and lower segmental branches (Fig. 1H) with a 5.5 × 30 Ever-Cross, a 6 × 30 Ever-Cross, an 8 × 20 Ever-Cross, and a 9 × 60 POWERFLEX sequentially with acceptable final results (Fig. 1I) and without any complications.

**Table 1:** Right heart catheterization data

First patient	Systemic blood pressure	RV pressure	Mean RA pressure	Mean PAP	Pulmonary vascular resistance	O_2_ saturation
	90/60 mmHg	120/30 mmHg	30 mmHg	66 mmHg	21 WU	85%
Second patient	**Systemic blood pressure**	**RV pressure**	**Mean RA pressure**	**Mean PAP**	**RPA pressure distal to the stenosis**	O_2_ **saturation**
	120/70 mmHg	50/10 mmHg	10 mmHg	26 mmHg	18 mmHg	94%
Third patient	**Systemic blood pressure**	**RV pressure**	**Mean RA pressure**	**Mean PAP**	**RPA pressure distal to the stenosis**	**LPA pressure distal to the stenosis**
	100/60 mmHg	75/20 mmHg	20 mmHg	45 mmHg	25/10 mmHg	20/10 mmHg

*LPA* left pulmonary artery, *PAP* pulmonary artery pressure, *RA* right atrium, *RPA* right pulmonary artery, *RV* right ventricle

The intervention was repeated in another session 2 months later. In the second session, a selective RPA injection in the anteroposterior projection demonstrated good results for the previous procedure. Afterward, with the aid of a long sheath and a JR catheter, the left lower branch of the LPA, which was bifurcated, was wired. Next, BPA was performed with a 3 × 15 Accuforce, a 4.5 × 15 NC TREK, and a 6 × 60 POWERFLEX with good results (Fig. 1J, K, and L). Finally, BPA was carried out on the upper branch with a 4.5 × 15 NC TREK with admissible results and without any complications (Fig. 1M and N).

The final hemodynamic study showed a significant reduction in systolic pulmonary artery pressure (PAP) (from 120 to 60 mmHg).

### Case 2

**Fig. 2:**
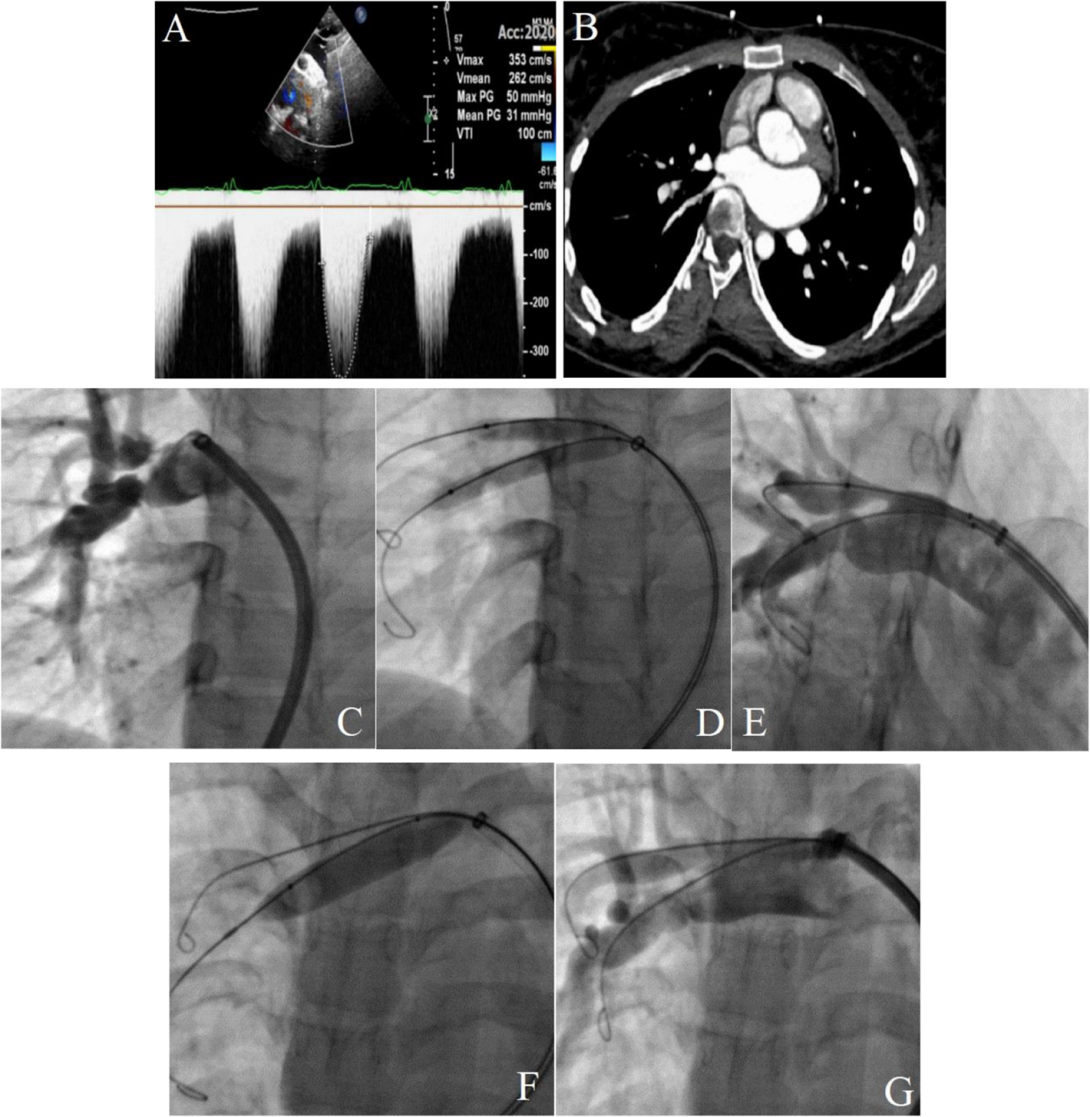
(**A** and **B**) Transthoracic echocardiography depicts a significant gradient at the origin of the right pulmonary artery (**A**). Pulmonary computed tomography angiography reveals a significant stenosis at the origin of the right pulmonary artery (**B**). (**C**, **D**, **E**, **F**, and **G**) Selective right pulmonary artery injection shows significant stenosis in the upper and lower lobar branches (**C**). Kissing balloon dilation of both branches is done (**D**). Injection after balloon percutaneous angioplasty shows residual stenosis in both branches (**E**). Separate and simultaneous balloon percutaneous angioplasty procedures are performed on both branches using larger balloons (**F**). Final angiography shows mild residual stenosis in the lower branches with acceptable blood flow in the distal branches (**G**)

A 24-year-old woman was referred to us with a complaint of exertional dyspnea (New York Heart Association functional class II-III) and atypical chest pain with occasional palpitation. In terms of general appearance, no abnormal features were notable. On physical examination, the patient had normal blood pressure and normal S1 and S2; in addition, a grade II systolic murmur was audible at the right sternal border without trills. No evidence of cyanosis or clubbing was found. Electrocardiography showed an incomplete right bundle branch block. In chest X-ray, right-sided lung vascularity in the upper and middle zones was obviously reduced. In echocardiography, the LV had normal size and function, while the RV was mildly enlarged with mild dysfunction. No evidence of right ventricular outflow tract (RVOT) obstruction (subvalvular, valvular, or supravalvular PA stenosis) was reported. Significant turbulence at the origin of the RPA with a peak pressure gradient of 50 mmHg was marked (Fig. 2A). The pulmonary arteries were accurately assessed with CTA, which showed a significant stenosis at the origin of the RPA (Fig. 2B).

According to the patient’s symptoms, history, and imaging findings, she was scheduled for RHC, selective pulmonary angiography, and probably interventional procedures.

#### Right heart catheterization data (Table 1)

##### Procedural technique

Under local anesthesia, hemodynamic monitoring, and complete intravenous heparinization, vein sheaths (6 F) were inserted into the right femoral artery. A selective LPA injection showed normal vascularity with no stenosis, whereas a selective RPA injection through a long delivery sheath (Cook 8 F) and a multipurpose catheter demonstrated a significant stenosis in the RPA just after the MPA bifurcation (right upper and lower lobar arteries) (Fig. 2C). The right upper and lower lobar arteries were wired with hydrophilic wires; then, they were replaced with Amplatz Super-Stiff guidewires. Afterward, simultaneous BPA was performed on both branches with a 7 × 40 Armada and a 7 × 60 Armada (Fig. 2D). The post-BPA angiography showed residual stenosis in both branches (Fig. 2E). Consequently, BPA was performed on each branch separately with larger balloons (a 9 × 40 Ever-Cross and a 12 × 40 Optimed-Zelos), followed by simultaneous BPA on both branches. The final angiography revealed a mild residual stenosis in the lower branch with acceptable blood flow in the distal arteries (Fig. 2F). The final pressure study demonstrated a significant drop in the mean trans-pulmonary pressure gradient (RPA to MPA) (30 to 10 mmHg) without any complications.

### Case 3

A 49-year-old woman with a history of radiotherapy due to breast cancer several years earlier was referred with a complaint of exertional dyspnea (New York Heart Association functional class III), chest pain, and occasional hemoptysis. In terms of general appearance, no abnormality was detected. On physical examination, the patient had an oxygen saturation level of 80% in room air; elevated jugular venous pressure; a normal S1; a loud P2, which was prominent at the left sternal border and apex; and mid-systolic murmurs in the left and right second intercostal spaces. A chest X-ray revealed cardiomegaly, markedly diminished bilateral pulmonary vascularity, and a dilated MPA. Electrocardiography demonstrated a right axis deviation and RV hypertrophy with strain patterns.

In echocardiography, the LV had normal size but mild systolic dysfunction, and the RV was moderately-to-severely enlarged and moderately dysfunctional. Also demonstrated were moderate-to-severe tricuspid regurgitation (gradient = 70 mmHg) and moderate pulmonary insufficiency with no RVOT obstruction (subvalvular, valvular, or supravalvular PA stenosis). A turbulent flow at the origin of both RPA and LPA was notable. In addition, moderate-to-severe circumferential pericardial effusion with no criteria of physiologic tamponade and a stretched patent foramen ovale (6 mm) with a net right-to-left shunt and without any other anomalies were depicted.

**Fig. 3:**
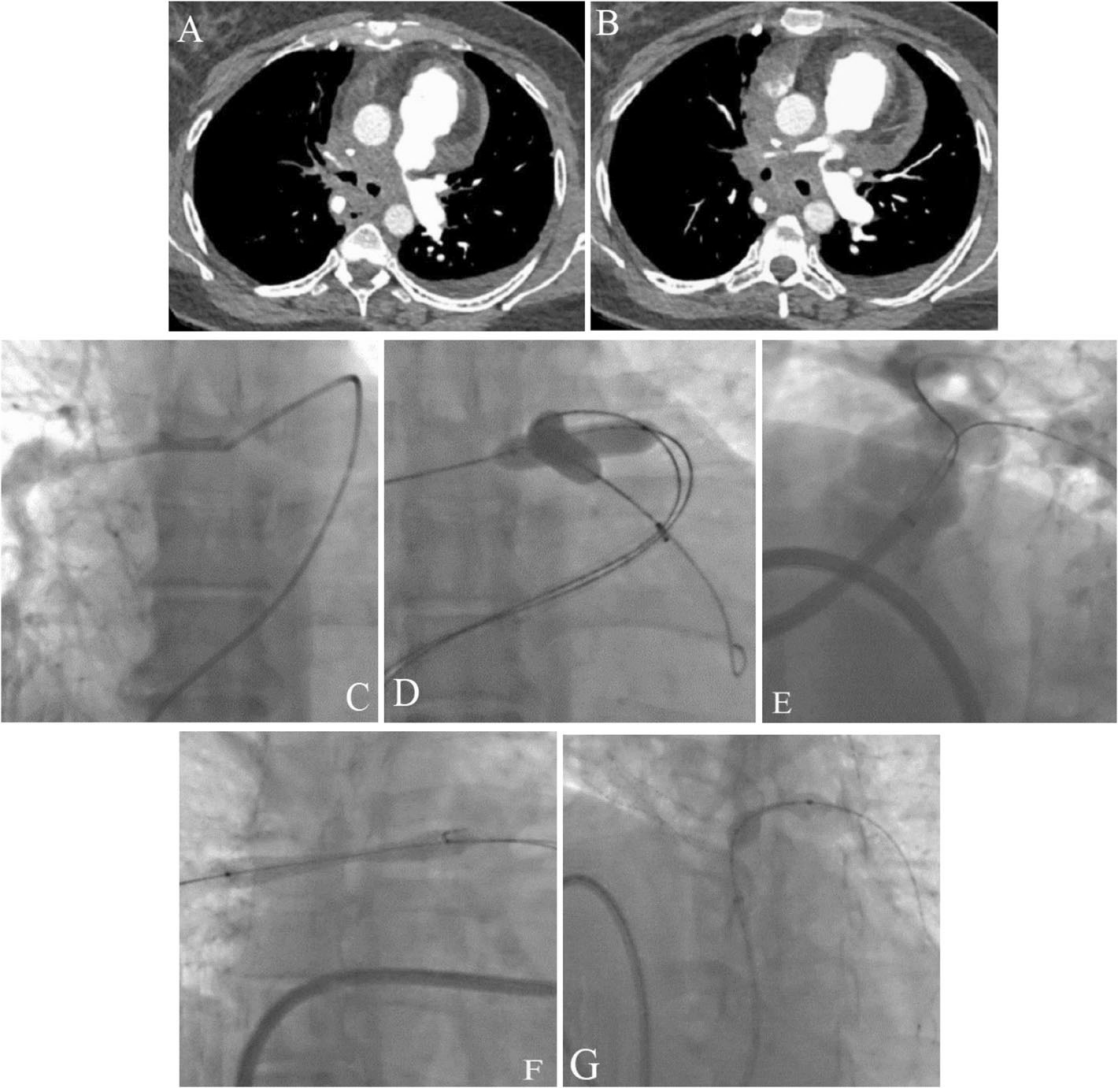
(**A** and **B**) The images reveal significant stenosis at the origins of the left and right pulmonary arteries as well as massive pericardial effusion. (**C**, **D**, **E**, **F**, and **G**) Selective right pulmonary artery injection shows a significant diffuse stenosis at the origin (**C**). Pre-dilation of both right and left pulmonary arteries is done by the balloon swallowing technique, and the balloons are advanced further (**D**). Significant residual stenosis in both branches is delineated after balloon percutaneous angioplasty (**E**). Stenting of the right pulmonary artery is done, and the central waist of the stenosis is visualized (**F**). The central waist disappears completely, and the flaring of the proximal edge of the stent is done. Afterward, stenting of the left pulmonary artery is performed. The central waist of the lesion is illustrated (**G**)

For further evaluation, a pulmonary CTA was performed. It reported a significant stenosis at the origin of both LPA and RPA just after the bifurcation (Fig. 3A and B).

Accordingly, the patient was scheduled for RHC, selective pulmonary angiography, and probably interventional procedures.

#### Right heart catheterization data (Table 1)

The selective coronary angiography depicted a significant tubular stenosis in the ostio-proximal part of the left anterior descending coronary artery.

##### Procedural technique

Under local anesthesia and following RHC, a selective MPA injection showed a significant diffuse stenosis in the RPA and a focal stenosis in the LPA just after the bifurcation. The selective RPA injection is shown in Fig. 3C. Through a long delivery sheath and a multipurpose catheter, the RPA and the LPA were wired with hydrophilic wires. Next, the wires were replaced with Amplatz Super-Stiff guidewires. By several attempts and the balloon-swallowing technique, the long delivery sheath and balloons were advanced toward the distal part of both branches (Fig. 3D). Initially, BPA was performed on the RPA and the LPA with a 9 × 40 ev3 and a 10 × 40 Oceanus (Fig. 3D). A subsequent injection illustrated a significant residual stenosis in both branches (Fig. 3E). Thus, in the first instance, by repetitive angiography, the correct position of the stent was confirmed and the RPA was stented with a 10 × 58 ivascular Stent and post-dilated with a 14 × 40 Atlas Gold (Fig. 3F). Afterward, the LPA was stented with a 10 × 40 Formula and post-dilated with a 12 × 40 Conquest (Fig. 3G) with no residual stenosis or gradient and with optimized results in the final angiography.

In another session, a percutaneous coronary intervention was performed on the ostio-proximal part of the left anterior descending coronary artery.

## Discussion

In pediatric patients suffering from PPAS, several randomized trials have reported the long-term outcome of endovascular treatment. In contrast, in adult patients with PPAS, the literature contains a paucity of information and there are no definite therapeutic strategies. The majority of patients with PPAS have concomitant congenital heart diseases, a history of palliative surgical therapies during childhood, or syndromic characteristics. Acquired cases are rare, and they are frequently underestimated in adulthood and managed inappropriately.

### Diagnostic workup

A high index of suspicion during the initial evaluation of patients with PH is essential for the precise diagnosis and treatment of PPAS bearing in mind that the treatment for PPAS is BPA, not PH-specific therapies [5, 7].

Echocardiography is the first modality; it demonstrates the size and function of both ventricles. In patients suffering from significant PPAS, the enlargement and hypertrophy of the RV are prominent. This explains the importance of a prompt investigation to establish the etiology of the RV pressure overload, which could be RVOT obstruction (subvalvular, valvular, or supravalvular PA stenosis), any etiology of PH (according to the PH classification), intracardiac shunting, stretched patent foramen ovale, and concomitant anomalies such as supravalvular aortic stenosis in Williams–Beuren syndrome and the double-outlet right ventricle. Valvular assessment is mandated to determine the severity and gradient of tricuspid regurgitation, the severity of pulmonary insufficiency, and the mean PAP. Further, the PA branches should be visualized to assess the collaterals and to detect any turbulence.

Cardiac and pulmonary CTA is more accurate than echocardiography for the detection of the underlying causes of RV pressure overload. CTA confers a precise assessment of not only PPAS in terms of the number, location, and severity of the stenosis as well as the configuration and the diameter of the distal branches and collaterals but also CTEPH, pulmonary atresia, and RV-PA conduit patency.

Nuclear quantitative perfusion scanning is another diagnostic tool, which might show marked differences in radiotracer uptake between the lungs. Depending on the location and severity of the stenosis, lung perfusion scanning may reveal segmental defects or unbalanced quantitative perfusion in the lungs, which might lead to the misdiagnosis of CTEPH. Further assessment with other modalities and clinical history is, therefore, mandatory.

Angiography is still the gold standard for detailed imaging [8]. A selective PA injection can delineate the exact anatomy, especially in complex lesions at the lobar or segmental level. Catheterization studies with the measurement of pressure gradients across the constricted areas are also helpful. A sudden rise in systolic pressure (≥ 20 mmHg) in the MPA or a major branch during continuous pressure recording from the distal PA to the RV is highly suggestive of a significant PA stenosis. Furthermore, wide pulse pressure and deep dicrotic notching on the pressure tracing from the proximal PA are commonly noted. Therapeutic interventions can be done in the same session.

Table 2 presents a summary of the latest indications for the treatment of PPAS.

**Table 2:** Indications for the treatment of peripheral pulmonary artery stenosis [7, 9]

• A reduction in the vessel diameter of greater than 40% or a reduction in the vessel diameter of greater than 30% in cases with additional right ventricular volume overload (e.g., atrial septal defect, tricuspid regurgitation, and pulmonary regurgitation) • After cavo-pulmonary anastomoses (the Glenn or Fontan shunt), pulmonary artery stenosis should be considered for therapy even if the reduction in the vessel diameter is less than 30%. • Bilateral pulmonary artery stenosis resulting in a right ventricular systolic pressure of more than 50% of the systemic pressure • Significant stenosis with a measurable gradient of greater than 20 mmHg (30 mmHg) • Pulmonary artery branch stenosis resulting in a reduction in an ipsilateral lung perfusion level of less than 20% (30%) • Hypertension in other unobstructed pulmonary artery segments	

### Therapeutic considerations

For many years, PPAS was surgically treated during the repair of other anomalies; nevertheless, the long-term result was far from satisfactory because the surgical accessibility of peripheral stenosis was suboptimal. The past 2 decades have witnessed a rise in the utilization of endovascular approaches to the treatment of this group of patients, and surgical repair has been limited to patients with proximal stenosis [10, 11]. Congenital or acquired forms of PPAS (isolated or post-palliative surgery) are frequently multiple lesions that are bilateral and non-uniform. Such stenotic forms can involve the central, bifurcation, lobar, and segmental arteries.

The first option for dilating stenotic arteries in adults is BPA, whereby the most stenotic segment is dilated first with a balloon inflation time of 10 to 60 s depending on the response of the waist. The balloons are inflated 2 or 3 times. The balloon size is chosen 2 to 6 times greater than the narrowest diameter of the treated stenotic vessel segment or 1.5 times greater than the diameter of the surrounding pre- or post-stenotic vessel segments [12, 13]. Different types of balloons are available: high pressure, low pressure, balloon-in-balloon, and scoring. In trials, the success rate of primary BPA is reported to range between 60 and 70%. Recurrent stenosis is not uncommon as could occur in approximately 35% of successfully dilated vessels [14]. In failed BPA procedures, alternative balloons such as high-pressure balloons or scoring balloons might be effective. Stent placement in the central pulmonary arteries has been performed with success, while stenting of the small segmental arteries has been less successful. The primary cardiac diagnosis of post-BPA patients with restenosis leading to a redo-intervention (re-dilation after primary BPA or secondary stenting) is mainly dextro-transposition of the great arteries. Additionally, restenosis following BPA could occur after tetralogy of Fallot repair, at the site of Blalock-Taussig or Waterston shunts, or as a result of the operator’s inability to advance the catheter across the stenosis [14]. It seems that there is no correlation between restenosis and the balloon-to-vessel ratio or the pressure of inflation, although some believe that post-BPA restenosis may be influenced by vessel trauma induced by higher pressure during dilation and a high balloon-to-vessel diameter ratio.

Table 3 offers a summary of the criteria for the success of PPAS management. We followed up our 3 patients by TTE at 6 months after the procedure and then yearly by TTE if they were asymptomatic.

**Table 3:** The goal of the treatment (criteria for success) [15]

• Relief of pulmonary artery stenosis or a minimum increase in the vessel diameter of 50% with improvements in the angiographic flow in the dilated pulmonary artery segment (angiographic success) • Reduction in the right ventricular systolic pressure to less than 50% of the systemic pressure or a decrease in the right ventricular systolic pressure by 30% or more • A minimum reduction of 50% in the distal pulmonary artery to the main pulmonary artery peak systolic pressure gradient • Improvement in the ipsilateral lung perfusion level (> 35%) • Improvement in the peripheral lung perfusion, ameliorating the hemodynamic effects of pulmonary valve regurgitation	

Our first patient was a case of arterial tortuosity syndrome with bilateral multiple lobar and segmental stenosis. We performed BPA on all the stenotic sites in 2 sessions with minimal residual stenosis within the first year of the procedure. Our default strategy for balloon size selection in PPAS is a balloon diameter-to-average proximal and distal vessel diameter (pre- and post-stenotic vessel segments) ratio of 1.1/1 given that we use high-pressure balloons. Our second patient was a case of isolated PPAS, for which we performed kissing balloon angioplasty on the upper and lower RPA lobar branches with successful results according to CTA and TTE at 2 years’ follow-up.

An alternative option for the treatment of PPAS is primary stenting or bailout stenting. Primary stenting is usually performed on proximal stenosis, where the stent-to-vessel diameter ratio does not usually exceed the diameter of the vessel adjacent to the stenotic segment. In pediatric trials, the success rate of stenting is comparable to that of BPA and is approximately 70 to 80% [14, 15]. Primary stenting is used as the method of choice for the treatment of stenosis that seems to be untreatable by BPA alone due to immediate post-intervention elastic recoil, non-dilatable fibrotic lesions, or external compression. Secondary stenting is performed when primary BPA fails with several types of stents including self-expandable and balloon-expandable types.

Our third patient was a case of radiation-induced PPAS. Because primary BPA on both the LPA and the RPA (fibrotic lesions that were BPA-resistant and immediately recoiled despite several BPA attempts) proved unsuccessful, we carried out the stenting and post-dilating of both branches. Failed stenting is not uncommon in infancy, and frequent stent re-dilation is needed with the somatic growth of children. The long-term outcomes of stenting in adults are not well known, and further research is warranted for clarification.

### Complications

A possible early unfavorable result of stenting is acute pulmonary edema, defined as a sudden elevation in the perfusion of downstream (unconditioned) pulmonary vessels and the resultant intense rise in the distal PA pressure [16, 17]. The etiology and indicators of reperfusion pulmonary edema after PA angioplasty have yet to be elucidated; nevertheless, it has been hypothesized that a more prominent increase in the vessel diameter and the distal PA pressure after ballooning might trigger more significant reperfusion injury [18, 19]. Another issue that mandates intensive surveillance after stenting is in-stent restenosis, the incidence of which in most trials on pediatric and adolescent populations is reported to be about 15 to 35% at 5 years’ follow-up. These findings are a testament to the vital importance of follow-ups in adult patients. Other rare but devastating complications of stenting are wire or balloon-induced vessel dissection, perforation, and aneurysmal formation. Some believe that aneurysm formation is not associated with the balloon-to-artery ratio or high-pressure inflation but that it occurs mostly as a result of inflation in small branches distal to the stenosis [19, 20]. However, we urge that appropriate balloon or stent selection be accorded due significance.

## Conclusions

PPAS is a rare congenital or acquired phenomenon whose misdiagnosis or inappropriate treatment is not unusual. Therefore, a high index of suspicion is required to distinguish patients who present with irreversible PH and damage. The main treatment strategy is the balloon dilation of the most stenotic segments. With respect to appropriate balloon size selection in PPAS, we recommend that the balloon diameter-to-average proximal and distal vessel diameter (pre- and post-stenotic vessel segments) ratio be considered 1.1/1. Some investigators believe that high-pressure inflation could lead to more endothelial damage, increasing the chance of restenosis or aneurysmal formation. Given that in most patients, the stenotic segments are frequently bilateral and multiple, BPA is usually performed in several sessions. In those with the involvement of more proximal parts (i.e., the main PA or the bifurcated site) or those who do not respond to BPA, scoring balloons and stenting are alternative options. Considering the lack of long-term outcome trials on adult patients, the long-term monitoring of these patients by noninvasive imaging modalities is highly recommended to determine the best therapeutic approaches.

## Data Availability

All data are available for more consideration.

## Abbreviations

PPAS: Peripheral pulmonary artery stenosis
PH: Pulmonary hypertension
CTEPH: Chronic thromboembolic pulmonary hypertension
RV: Right ventricle
RPA: Right pulmonary artery
LPA: Left pulmonary artery
BPA: Balloon pulmonary artery angioplasty
RVOT: Right ventricular outflow tract
TTE: Transthoracic echocardiography
TEE: Transesophageal echocardiography
